# Hemp-Derived Cellulose/NiFe_2_O_4_ Biocomposite Hydrogels for Flexible Optoelectronics

**DOI:** 10.1021/acsomega.6c04724

**Published:** 2026-10-07

**Authors:** Orhan Yalçın, Ahmed M.J. Al-dala Ali, Ramazan Coşkun, Hasan Uslu

**Affiliations:** † Faculty of Sciences, Department of Physics, Niğde Ömer Halisdemir University, 51240 Niğde, Türkiye; ‡ Institute of Science, Niğde Ömer Halisdemir University, 51240 Niğde, Türkiye; § Department of Chemistry, 162338 Yozgat Bozok University, Yozgat 66500, Türkiye; ∥ Department of Food Engineering, Niğde Ömer Halisdemir University, 51240 Niğde, Türkiye

## Abstract

This study presents a systematic investigation into the optical and broadband dielectric behaviors of sustainable biocomposite hydrogels, synthesized from hemp biomass-derived cellulose and a poly­(AMPS/IA) network reinforced with varying concentrations of NiFe_2_O_4_ nanoparticles, hereafter denoted as NiFe_2_O_4_ biocomposite hydrogels (NiFH). The primary objective was to determine the suitability of these eco-friendly materials for flexible optoelectronic applications by elucidating the influence of filler loading on charge transport mechanisms. Structural and morphological characterizations via X-ray diffraction (XRD), scanning electron microscopy (SEM), and scanning transmission electron microscopy (STEM) confirmed the successful formation of a densely cross-inked amorphous matrix. The dispersion of the inorganic phase, without severe microscale agglomeration, was evidenced by the electron-dense domains resolved in the STEM images, while the characteristic spinel-type metal–oxygen vibrations identified by FTIR were consistent with the retention of the NiFe_2_O_4_ ferrite phase. Optical characterization revealed that the hydrogels exhibit tunable semiconductor properties, with direct band gap energies (*E*_*g*_^*d*^) modulated between 2.75 and 3.57 eV. While the NiFH2 formulation (0.5% NiFe_2_O_4_) showed the highest structural order, increasing the NiFe_2_O_4_ content to 1.0% (NiFH4) induced localized interfacial disorder, as evidenced by an elevated Urbach energy and a corresponding narrowing of the forbidden gap. Broadband dielectric spectroscopy demonstrated that the frequency-dependent response is governed by Maxwell–​Wagner–Sillars interfacial polarization and Koops’ phenomenological models. Unlike conventional trends, the NiFH4 sample emerged as the optimal formulation, exhibiting peak dielectric permittivity and enhanced parallel capacitance due to the expanded electroactive surface area. This impedance analysis conclusively demonstrated that charge carriers encounter their primary impedance barriers at the microstructural interfaces and grain boundaries, which consistently exhibit significantly higher resistance than the bulk material. Moreover, increasing the NiFe_2_O_4_ concentration systematically diminished these internal resistances, thereby simultaneously enhancing both bulk and interfacial conductivity. The modeling further elucidated the capacitive dynamics of the system, revealing strong nonideal capacitive effects driven by interfacial polarization at low frequencies, which transitioned into highly stable dielectric behavior at higher frequencies. Ultimately, the synergistic combination of biomass-derived cellulose and magnetic nanoparticles positions these hydrogels as highly promising candidates for high-performance “green” energy storage systems and flexible bioelectronic sensing platforms.

## Graphical abstract

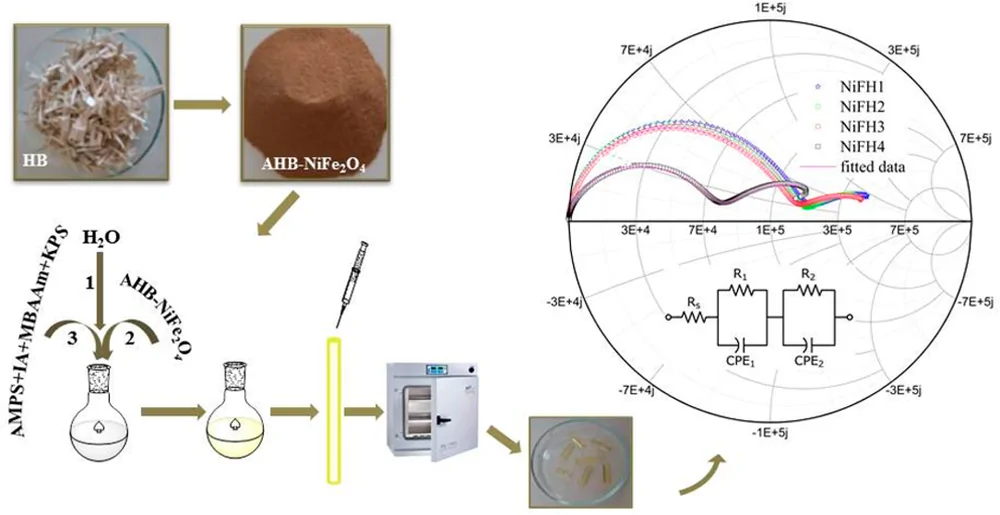

## 1 Introduction

The rapid proliferation of electronic waste has necessitated a paradigm shift toward sustainable, biodegradable alternatives to petroleum-derived plastics in the manufacturing of electronic components. Cellulose, recognized as the world’s most abundant renewable polymer, has emerged as a critical material in this transition due to its inherent thermal stability, mechanical robustness, and recyclability.^1,2^ Among the various cellulose sources, industrial hemp is particularly advantageous due to the high cellulose concentration in its bast fibers, short harvest cycles, and its favorable life-cycle assessment, which demonstrates a significantly lower environmental footprint compared to conventional crops.^3,4^ Beyond these sustainable attributes, hemp emerges as a leading candidate for “green electronics”, primarily due to the capacity of its fibers to form compatible matrices with functional fillers exhibiting distinct electrical and magnetic properties.^5,6^ This versatility extends its utility from traditional sectors, such as textiles and paper, to high-value-added fields including biomedical applications and electronic substrates.^3,5,7–10^

While pure cellulosic materials offer inherent sustainability, their application in advanced technologies often requires enhancement through composite engineering. Biocomposite materials, synthesized by reinforcing organic matrices with inorganic components (such as polysaccharides, metals, nanocarbons, and natural or synthetic polymers), exhibit mechanical, physical, and chemical properties superior to those of pristine biopolymers.^11^ Hemp-based biocomposites, in particular, represent a leading class within this new generation of eco-friendly materials, finding applications in construction panels, agricultural textiles, acoustic insulation, and high-strength flooring.^12–14^ However, recent research focuses increasingly on the functionalization of these composites for high-tech applications. Studies indicate that surface-modified cellulose fibers with enhanced hydrophilicity contribute to biodegradable, rigid, and high-strength architectures, with thermal and morphological properties that vary significantly based on structural composition.^15,16^

Extensive research has been dedicated to elucidating the optical, electrical, and dielectric characteristics of these cellulose-based architectures for use in flexible electronics and energy storage platforms.^17–19^ For instance, composite systems incorporating bismuth sodium titanate into oxidized cellulose films display semiconducting behavior, positioning them as promising candidates for next-generation optoelectronic devices. Similarly, cellulose matrices reinforced with sodium silicate nanoparticles and varying polypyrrole content have demonstrated that structural modifications can significantly influence morphological and dielectric parameters.^20–22^

In parallel with these advancements in solid-state composites, hydrogels have garnered significant interest as a distinct class of soft materials essential for flexible electronics. As three-dimensional, cross-linked polymer networks capable of retaining large amounts of intrinsic water, they exhibit structural similarity to biological tissues.^23–25^ Crucially, their exceptional biocompatibility allows for direct interfacing with the human body without inducing adverse physiological reactions, making them indispensable for medical and wearable applications. Capitalizing on these unique properties, recent developments have demonstrated that ultra-flexible triboelectric sensors incorporating liquid-metal and high-performance hydrogel networks can be seamlessly integrated into tactile interface platforms. Complementary studies have further explored these materials in wearable rehabilitation systems and glove-integrated human–machine interfaces, highlighting their distinct advantages for continuous physiological monitoring and digital health tracking.^23–25^

To transform these flexible hydrogel systems from passive substrates to active electronic components, the integration of magnetic spinel ferrite nanoparticles, such as nickel ferrite (NiFe_2_O_4_), offers a strategic solution. The incorporation of NiFe_2_O_4_ yields hybrid architectures that synergistically combine the mechanical compliance and biocompatibility of the cellulose–hydrogel network with the robust electromagnetic functionality of ferrites.^26^ Recent investigations indicate that dispersing NiFe_2_O_4_ within polymer matrices can substantially enhance dielectric permittivity, magnetic permeability, and optical absorption while preserving processability.^27,28^ In such systems, concentration-dependent behavior is particularly critical; nanoparticle loading directly modulates percolation networks, interfacial polarization, and charge transfer mechanisms, thereby enabling precisely tunable optoelectronic responses essential for advanced sensor and circuit applications^29^

However, despite the growing body of literature on cellulose composites and hydrogel sensors individually, a systematic investigation into doped hydrogels that integrate hemp-derived cellulose support with magnetic nanoparticles within a synthetic polymer network remains unexplored. This study addresses this gap by evaluating the optoelectronic potential of biocomposite hydrogels reinforced with NiFe_2_O_4_-modified cellulose (NiFe_2_O_4_–cellulose) and encapsulated within a poly­(2-acrylamido-2-methyl-1-propanesulfonic acid/itaconic acid) (poly­(AMPS/IA)) matrix. Specifically, this work characterizes the comprehensive structural, thermal, morphological, optical, and dielectric properties of these hydrogels under varying filler concentrations to elucidate underlying charge transport mechanisms. A distinguishing novelty of this research lies in the experimental construction and interpretation of Smith charts (typically reserved for RF engineering) to model the impedance characteristics of biocomposite systems equivalent to RC circuits. By demonstrating a strong correlation between experimental data and theoretical models, this study presents critical evaluation criteria for the applicability of NiFe_2_O_4_–​cellulose/poly­(AMPS/IA) hydrogels as functional elements in next-generation optoelectronic circuits.

## 2 Materials and Methods

### 2.1 Materials

The hemp plants cultivated at Yozgat Bozok University were provided by the University’s Hemp Research Institute, and the hemp biomass (HB) was obtained following the extraction of their fiber components. All chemical reagents were of analytical grade, purchased from Merck, and used as received without any further purification. These chemicals included iron­(III) chloride hexahydrate (FeCl_3_·6H_2_O), nickel­(II) chloride hexahydrate (NiCl_2_·6H_2_O), 2-acrylamido-2-methyl­propane­sulfonic acid (AMPS), itaconic acid (IA), *N*,*N*′-methyl­ene­bis­(acryl­amide) (MBAAm), and potassium persulfate (KPS), as well as concentrated sulfuric (H_2_SO_4_) and nitric (HNO_3_) acids.

### 2.2 Preparation of (NiFe_2_O_4_–Cellulose) Biocomposite Covered by Poly­(AMPS/IA) Hydrogel

Cellulose-supported nickel ferrite nanoparticles (NiFe_2_O_4_–cellulose) were synthesized following the procedure outlined in our previous study.^30^ To prepare the composite hydrogels, aqueous dispersions (5 mL) containing 1.0%, 0.75%, 0.5%, and 0.25% (w/v) NiFe_2_O_4_–cellulose were initially prepared by sonicating the relevant quantities of nanoparticles in distilled water. Subsequently, stoichiometric amounts of the monomers 2-acrylamido-2-methyl­propane­sulfonic acid (AMPS) and itaconic acid (IA), the cross-linker *N*,*N*′-methyl­ene­bis­(acryl­amide) (MBAAm), and the initiator potassium persulfate (KPS) were added to each dispersion. The mixtures were vortexed until homogeneous solutions were obtained.^31^ The resultant solutions were transferred into cylindrical pipet molds, sealed, and placed in an oven at 55 °C for 24 h to facilitate free-radical polymerization. Upon completion of the polymerization, the composite hydrogels were removed from the molds and washed repeatedly with excess distilled water to eliminate any unreacted residues. Finally, the samples were dried in an oven at 55 °C until a constant weight was achieved. The resulting NiFe_2_O_4_ biocomposite hydrogels (NiFH) containing 0.25%, 0.5%, 0.75%, and 1.0% NiFe_2_O_4_–cellulose were designated as NiFH1, NiFH2, NiFH3, and NiFH4, respectively. The preparation scheme is illustrated in detail in Figure 1.

**Figure 1:**
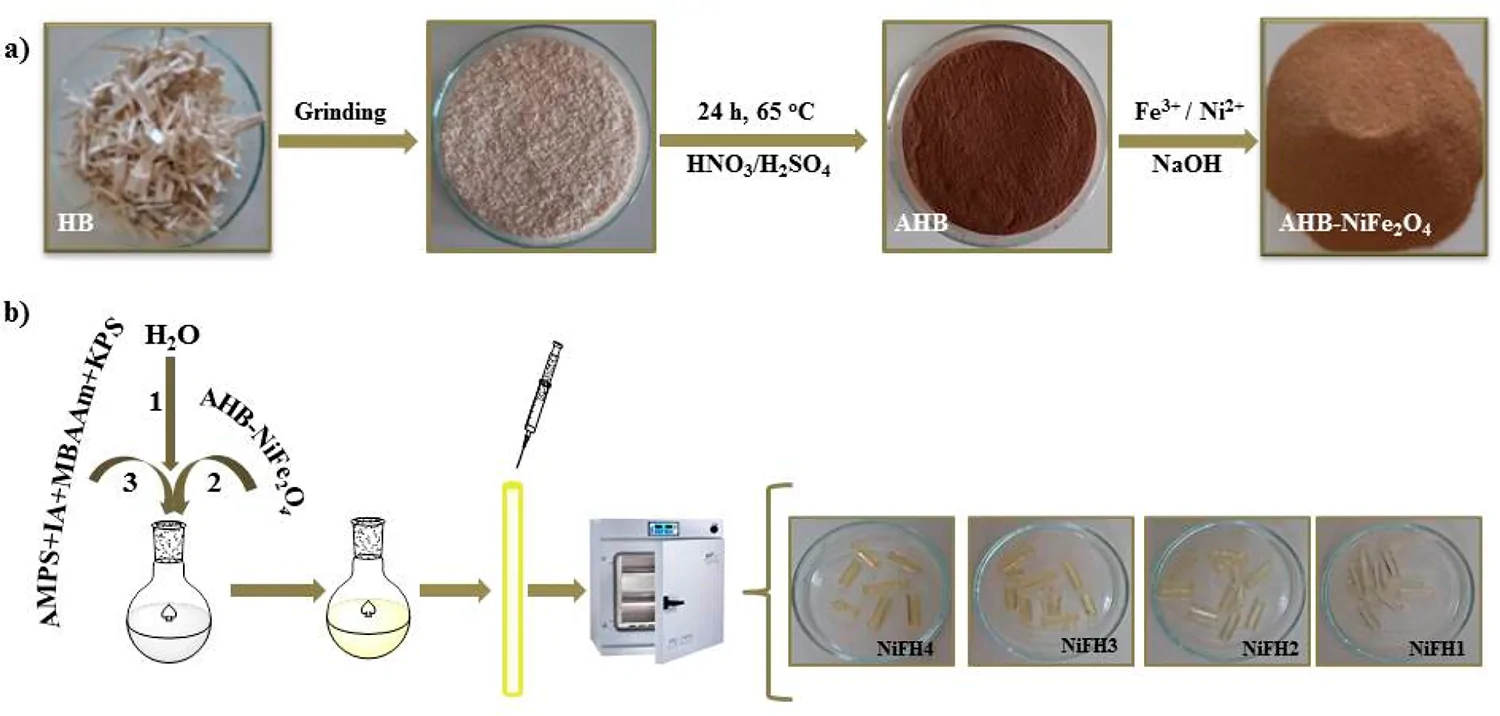
Schematic illustration of the synthesis route for (a) the cellulose-supported NiFe_2_O_4_ biocomposite and (b) the poly­(AMPS/IA) composite hydrogels.

### 2.3 Structural, Morphological, Optical, Thermal, and Dielectric Measurements

The crystalline structure of the cellulose-supported nickel ferrite nanoparticles was analyzed using X-ray diffraction (XRD) on a PANalytical Empyrean diffractometer equipped with Cu Kα radiation, operating at 45 kV and 40 mA over a 2θ range of 5°–90°. The surface morphology and elemental distribution of the NiFe_2_O_4_–cellulose-based hydrogels were examined using a ZEISS EVO 40 scanning electron microscope (SEM) equipped with an energy-dispersive X-ray spectroscopy (EDS) module for elemental mapping. Additionally, detailed microstructural and electronic configuration analyses were performed using a ZEISS Gemini 500 field emission scanning electron microscope (FESEM) operating in scanning transmission electron microscopy (STEM) mode. The optical properties of the hydrogels were characterized using an A360 spectrophotometer across a wavelength range of 190–900 nm at room temperature, employing quartz cuvettes with a 1 cm path length. The recorded absorbance spectra were used to determine the optical band gap energies (*E*_*g*_) using Tauc’s relation: *αhν* = *A*(*hν* – *E*_*g*_)^*n*^, where α is the absorption coefficient, *hν* is the photon energy, *A* is an energy-independent constant, and *n* depends on the nature of the electronic transition. The values of *n* were taken as 1/2 for direct allowed transitions and 2 for indirect allowed transitions. Consequently, the direct and indirect band gaps were extracted by extrapolating the linear regions of (*αhν*)^2^ versus *hν* and (*αhν*)^1/2^ versus *hν* plots, respectively. Furthermore, the Urbach energy (*E*_*U*_), representing the width of the localized states (band tails) within the forbidden gap, was calculated from the inverse slope of the ln­(α) versus *hν* plot. The thermal stability of the samples was assessed by thermogravimetric analysis (TGA) using a Hitachi STA 7300 analyzer under a nitrogen atmosphere, over a temperature range of approximately 25–800 °C. Dielectric and impedance measurements were conducted at room temperature using a Fytronix Dielectric Measurement System via the parallel plate capacitor method. The cylindrical hydrogel samples, having a thickness of approximately 1 mm, were sandwiched between two electrodes to facilitate electrochemical impedance spectroscopy (EIS). Data acquisition was performed under an applied AC voltage of 500 mV within a frequency range of 10 Hz to 10 MHz. To ensure data accuracy and eliminate external electromagnetic interference, all measurements were conducted inside a Faraday cage. The system’s proprietary software was utilized to extract complex impedance parameters, which were subsequently used to generate Smith charts and model the equivalent circuits derived from the Nyquist (complex impedance plane) plots.

## 3 Results and Discussion

### 3.1 Structural, Morphological, Chemical, and Thermal Characterization

Figure 2 presents a comprehensive characterization that tracks the stepwise structural, morphological, chemical, and thermal evolution of the biocomposite along its synthesis route, comparing three representative stages: the acid-activated hemp biomass (AHB), the intermediate NiFe_2_O_4_–AHB hybrid, and the final poly­(AMPS/IA) hydrogel network (NiFH4). The figure comprises SEM images of the three stages (Figure 2a–c), the corresponding STEM images (Figure 2d–f), the FTIR spectra (Figure 2g), the XRD pattern of NiFH4 (Figure 2h), and the thermogravimetric (TGA) weight-loss curves (Figure 2i). Taken together, these analyses indicate that the formation of the NiFH4 biocomposite is more accurately described as a hierarchical physicochemical assembly process than as a simple physical mixture, in which acid activation first generates hydroxyl-rich cellulose surfaces that anchor the NiFe_2_O_4_ nanoparticles, which subsequently become encapsulated within the growing poly­(AMPS/IA) network during in situ polymerization. The resulting hybrid interface is predominantly stabilized through hydrogen bonding, surface coordination, electrostatic interactions, and physical entanglement, producing homogeneous nanoparticle dispersion, efficient interfacial contact, and a mechanically integrated three-dimensional polymer architecture. This synergistic interfacial organization is considered to contribute to the enhanced dielectric response, improved electrical conductivity, and reduced optical band gap observed for the final hydrogel.

**Figure 2:**
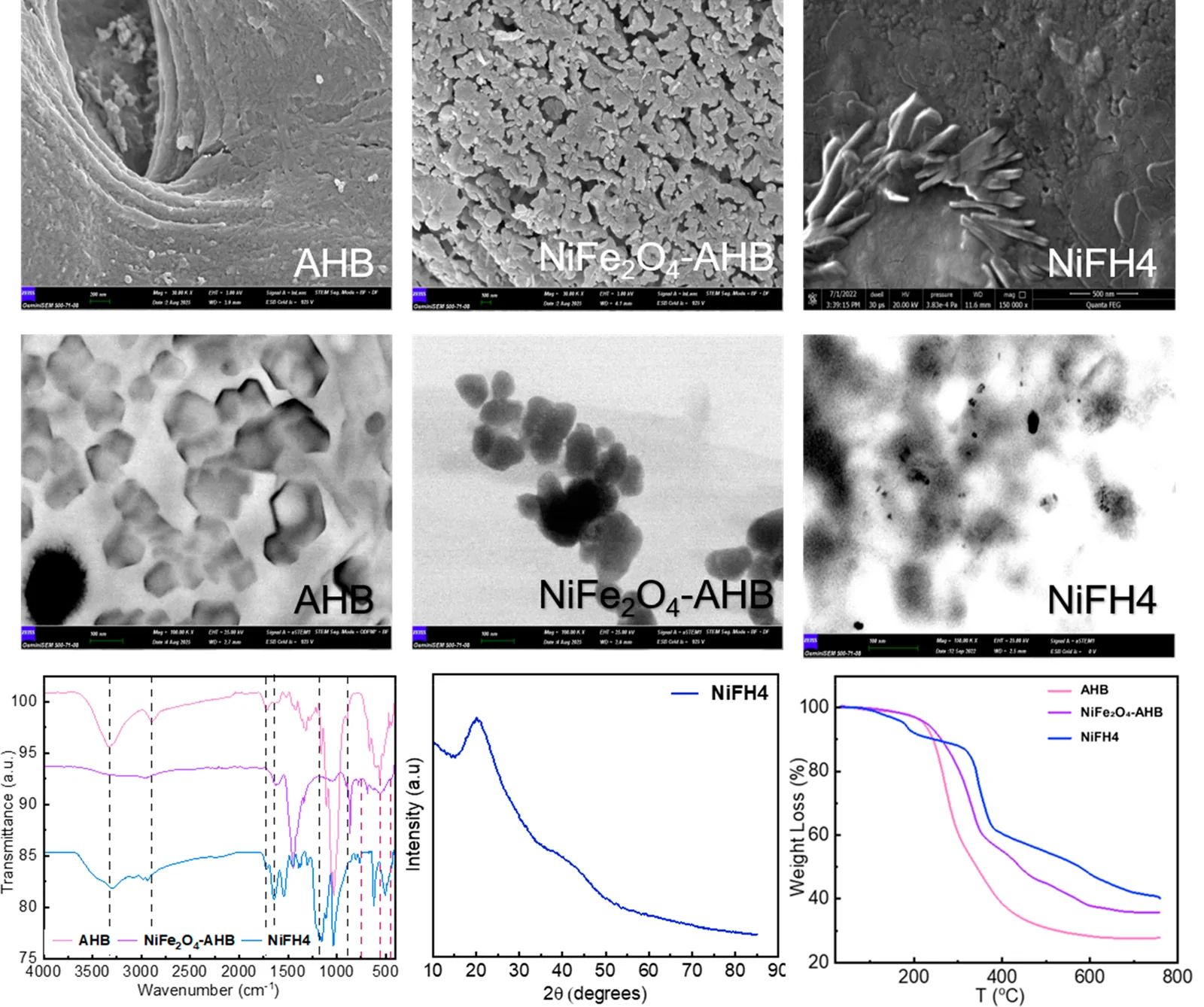
Structural, morphological, chemical, and thermal characterization illustrating the stepwise evolution of the NiFH4 biocomposite hydrogel: (a–c) SEM images of AHB, NiFe_2_O_4_–AHB, and NiFH4, respectively; (d–f) the corresponding STEM images, showing the homogeneous dispersion of electron-dense NiFe_2_O_4_ domains within the cellulose/poly­(AMPS/IA) matrix; (g) FTIR spectra of AHB, NiFe_2_O_4_–AHB, and NiFH4, confirming the coexistence of the characteristic cellulose, poly­(AMPS/IA), and NiFe_2_O_4_ functional groups; (h) XRD pattern of NiFH4; and (i) TGA weight-loss curves of AHB, NiFe_2_O_4_–AHB, and NiFH4.

The morphological evolution accompanying each synthesis stage is revealed by the complementary SEM and STEM analyses (Figure 2a–f). Following acid activation, the hemp-derived biomass exhibits a relatively smooth, lamellar morphology with a homogeneous matrix (Figure 2a), indicating the effective removal of noncellulosic components while the cellulose framework is preserved; the corresponding STEM image confirms a continuous, electron-transparent organic background (Figure 2d). The abundant hydroxyl groups exposed on this surface provide favorable sites for the immobilization of magnetic nanoparticles. Upon the incorporation of NiFe_2_O_4_, the cellulose surface becomes uniformly decorated with nanosized granular domains and develops a markedly rougher texture (Figure 2b), while the STEM image shows that the electron-dense NiFe_2_O_4_ nanoparticles are embedded within the cellulose matrix rather than existing as isolated clusters (Figure 2e). The absence of large micro-sized aggregates indicates that the cellulose framework effectively suppresses nanoparticle agglomeration and enlarges the effective interfacial area, which is expected to facilitate efficient stress transfer and charge transport across the hybrid.

After the in situ copolymerization of AMPS and itaconic acid, the NiFe_2_O_4_–AHB hybrid becomes encapsulated within a continuous three-dimensional hydrogel network. The SEM image of NiFH4 reveals a rough, interconnected architecture characterized by irregular, plate-like microstructures, typically smaller than 500 nm, that arise from the assembly of the primary NiFe_2_O_4_–cellulose units through interparticle forces and their robust interaction with the cross-linked poly­(AMPS/IA) network (Figure 2c).^34^ In the corresponding STEM image (Figure 2f), the heavier transition-metal-rich NiFe_2_O_4_ nanoparticles appear as dark, electron-dense regions distributed relatively homogeneously across the lighter polymeric background, without obvious phase segregation or secondary agglomeration. This dense encapsulation is driven by the robust intermolecular and intramolecular hydrogen bonding of the cellulose chains, which physically confines the nanoparticles and minimizes the free volume of the composite network.^32,33^ The preservation of homogeneous dispersion after polymerization demonstrates that the cellulose support effectively stabilizes the ferrite phase throughout hydrogel formation, thereby maintaining efficient interfacial contact within the final composite.

The FTIR spectra further demonstrate the stepwise structural evolution of the NiFH4 biocomposite, and all of the bands discussed below are individually labeled in Figure 2g. The broad absorption band centered at approximately 3400 cm^–1^ is assigned to the O–H stretching vibrations of cellulose hydroxyl groups and adsorbed water molecules. The band observed near 2920 cm^–1^ corresponds to the C–H stretching vibrations of the cellulose and polymer backbone.^36–39^ The absorption observed at approximately 1720 cm^–1^ is attributed to the carbonyl (C=O) groups originating from itaconic acid (IA) units, whereas the absorption band located at approximately 1650 cm^–1^ is attributed to the Amide I vibration (predominantly C=O stretching) of the amide groups originating from the AMPS units, while the band around 1545 cm^–1^ corresponds to the Amide II vibration associated with N–H bending coupled with C–N stretching.^29^ The presence of these characteristic acid and amide bands confirms the successful incorporation of IA and AMPS into the copolymeric hydrogel network. In addition, the characteristic sulfonic acid group of AMPS exhibits a strong absorption band at approximately 1190 cm^–1^, corresponding to the asymmetric stretching vibration of the SO_3_^–^ groups. The absorption band near 1040 cm^–1^ is assigned to the C–O–C stretching vibration of the cellulose backbone, while the characteristic β-glycosidic linkage is preserved at 893 cm^–1^,^40^ indicating that the cellulose framework remains structurally intact after the sequential modification process. Furthermore, the absorption bands observed at approximately 580 and 450 cm^–1^ are assigned to the metal–oxygen stretching vibrations of the tetrahedral and octahedral sites of the spinel NiFe_2_O_4_ structure,^30^ respectively, confirming the successful incorporation of magnetic nanoparticles into the cellulose/poly­(AMPS-*co*-IA) matrix. Collectively, these characteristic absorption bands provide strong spectroscopic evidence for the successful formation of the hierarchical NiFH4 biocomposite through the integration of cellulose, poly­(AMPS-*co*-IA), and NiFe_2_O_4_.

The crystallographic structure of the NiFH4 composite was examined by XRD (Figure 2h). The XRD results further support the successful structural integration. The characteristic diffraction peak of cellulose remains observable after the incorporation of NiFe_2_O_4_, while no additional crystalline reflections appear, indicating that no undesirable secondary or impurity phases form during composite synthesis. These observations demonstrate that the immobilization process preserves the cellulose framework while maintaining structural compatibility between the organic and inorganic phases without inducing any undesirable phase transformation. The distinct Bragg reflections of the NiFe_2_O_4_ nanoparticles are largely obscured by the dominant amorphous halo of the matrix, an effect that is structurally expected, given the low nanoparticle loading and the thick encapsulation of the inorganic phase within the dense hydrogel network.

Collectively, the FTIR, XRD, SEM, and STEM results consistently demonstrate that the interface among cellulose, NiFe_2_O_4_, and the poly­(AMPS/IA) network is governed predominantly by physicochemical interactions—namely hydrogen bonding, surface coordination, electrostatic interactions, and physical anchoring—which provide structural continuity across the hybrid interface, suppress nanoparticle aggregation, and ensure a uniform dispersion of the magnetic phase.^41,42^

Additionally, it can be said that the current STEM observations primarily provide morphological evidence of homogeneous nanoparticle distribution and close interfacial contact. Additionally, it should be noted that the present STEM observations primarily provide morphological evidence of homogeneous nanoparticle distribution and close interfacial contact. Furthermore, since cellulose is a semi-crystalline biopolymer while NiFe_2_O_4_ has a cubic spinel structure, classical epitaxial lattice matching is not expected in this hybrid system; the present discussion is therefore focused on the physicochemical interfacial interactions and structural evolution of the NiFe_2_O_4_–​cellulose/poly­(AMPS/IA) biocomposite hydrogel, supported by the available spectroscopic and microscopic evidence rather than direct atomic-resolution observations.

Finally, the thermal behavior of the samples provides additional insight into the structural robustness of the composite (Figure 2i). The residual mass increases progressively from AHB to NiFe_2_O_4_–AHB and, finally, to NiFH4, indicating a stepwise enhancement of thermal resistance at each synthesis stage. The incorporation of NiFe_2_O_4_ into the cellulose matrix delays thermal degradation and raises the char yield, suggesting that the ferrite phase acts as a thermally stable inorganic barrier that restricts polymer-chain mobility, while the NiFH4 hydrogel exhibits the highest residual mass and the most gradual weight-loss profile owing to the synergistic stabilization afforded by the three-dimensional poly­(AMPS/IA) network together with the uniformly dispersed NiFe_2_O_4_ domains. These trends are fully consistent with the SEM and STEM analyses discussed above. Although long-term cyclic durability and device-level operational stability were beyond the scope of the present work, the TGA results clearly demonstrate that the composite possesses a markedly superior resistance to thermally induced structural degradation compared with its precursor materials.

### 3.2 Elemental Composition and Spatial Distribution (EDS Analysis)

To further corroborate the chemical composition and evaluate the spatial distribution of the constituents, energy-dispersive X-ray spectroscopy (EDS) and elemental mapping were performed on the NiFH4 sample (Figure 3), and the corresponding EDS spectrum is provided in the Supporting Information (Figure S1). As expected from the predominantly organic nature of the biocomposite, the elemental maps reveal that carbon, oxygen, nitrogen, and sulfur dominate the structure (Figure 3e–h). The dense and homogeneous distribution of these elements confirms the uniform formation of the structural backbone, comprising the hemp cellulose and the cross-linked poly­(AMPS/IA) network. It should be emphasized, however, that EDS cannot resolve the inorganic phase in the final composite. The precursor is incorporated at concentrations of 0.25–1.0% (w/v) across the NiFH1–NiFH4 series, and the ferrite constitutes only around one-quarter of the precursor powder; even in NiFH4, the sample of highest loading examined here, the Fe and Ni contents of the hydrogel are of the order of 0.2–0.3 wt % and 0.1–0.2 wt %, respectively. These values lie at or below the practical detection limit of EDS for a millimeter-thick specimen composed almost entirely of light elements, where the bremsstrahlung background from the organic matrix is high and absorption effects are severe. Consistent with this, the Fe Kα and Ni Kα emission lines are not resolved above the background in the spectrum of the composite (Figure S1b), and the present EDS data are therefore reported as the elemental profile of the organic matrix rather than as evidence for the presence or distribution of the magnetic phase.

**Figure 3:**
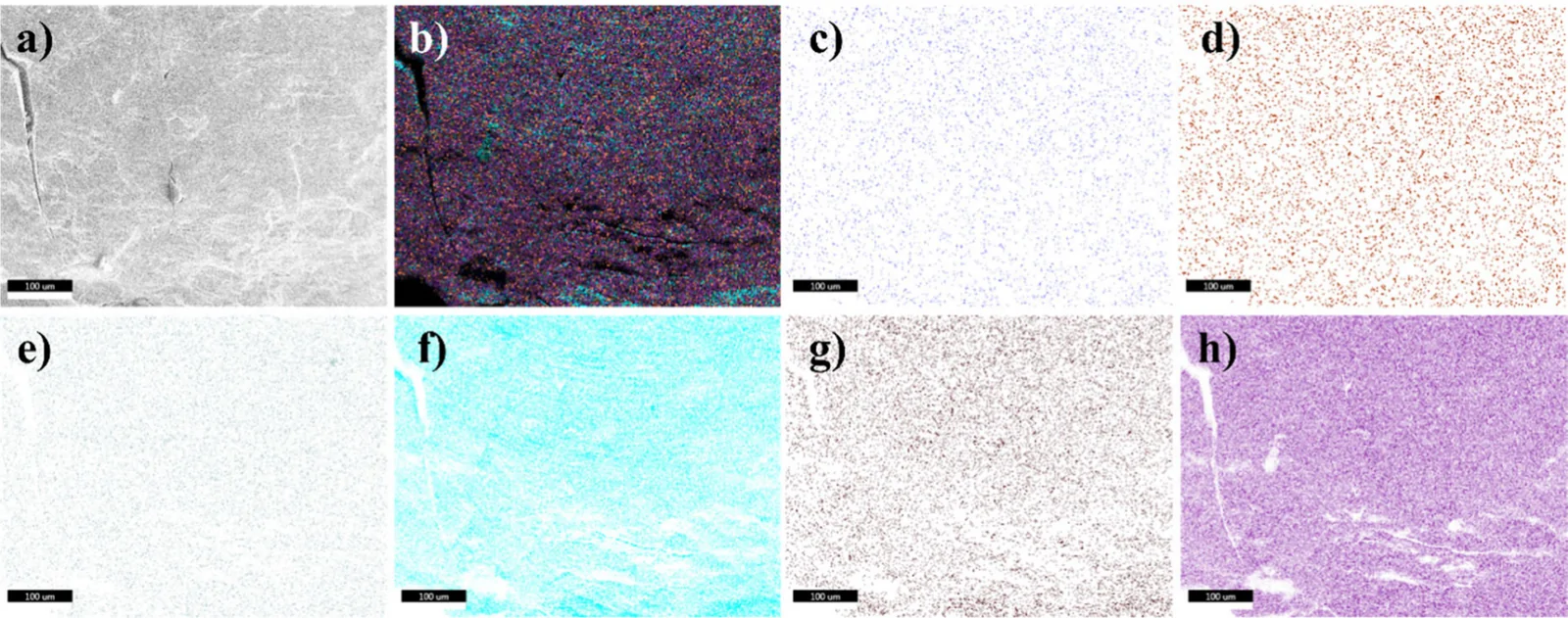
Energy-dispersive X-ray spectroscopy (EDS) analysis of the NiFH4 biocomposite hydrogel: (a) SEM micrograph of the selected surface area, (b) overlaid multielemental map, and individual spatial distribution maps for (c) Ni, (d) Fe, (e) S, (f) O, (g) N, and (h) C.

The composition of the inorganic phase was instead established at the precursor stage, before dilution within the hydrogel network. The EDS spectrum of the NiFe_2_O_4_–cellulose precursor (Figure S1a) resolves the Fe Kα (6.40 keV) and Ni Kα (7.48 keV) lines unambiguously above the bremsstrahlung continuum, yielding 12.27 wt % Fe and 8.09 wt % Ni with relative uncertainties below 1%. At this stage, the ferrite is present at a concentration well within the reliable working range of the technique. The retention of the spinel NiFe_2_O_4_ phase within the composite is further supported by the characteristic metal–oxygen vibrational bands identified in the FTIR analysis (Section 3.1), while its dispersion is evidenced by the electron-dense domains resolved in the STEM images (Figure 2f), which show no evidence of large aggregates. The dispersion of the inorganic fillers within the continuous hydrogel matrix, without extensive clustering, is a fundamental prerequisite for ensuring reliable and consistent optoelectronic and dielectric responses throughout the composite material.

### 3.3 Optical Properties

The UV–visible (UV–vis) absorption spectra of the NiFe_2_O_4_–cellulose biocomposite hydrogels, recorded over the wavelength range of 200–800 nm, are presented in Figure 4a. While minor absorption variations are observable below 400 nm, the overall optical response of all samples exhibits a similar frequency-dependent behavior that strictly conforms to the Beer–Lambert law.^43,44^ A careful examination of the spectra reveals that the maximum optical absorption occurs in the ultraviolet region (200–320 nm). Beyond this region, the absorbance decays exponentially with increasing wavelength before stabilizing at higher wavelengths. The significant absorption of photons in this specific UV wavelength range is primarily attributed to the intrinsic organic nature of the biopolymer matrix, as well as the structural modifications induced by the acid-activation process.^45^ Moreover, the optical response of the samples remained essentially unchanged when the measurements were repeated after approximately one year, confirming the long-term stability and reproducibility of the biocomposite hydrogels (Figure S2).

**Figure 4:**
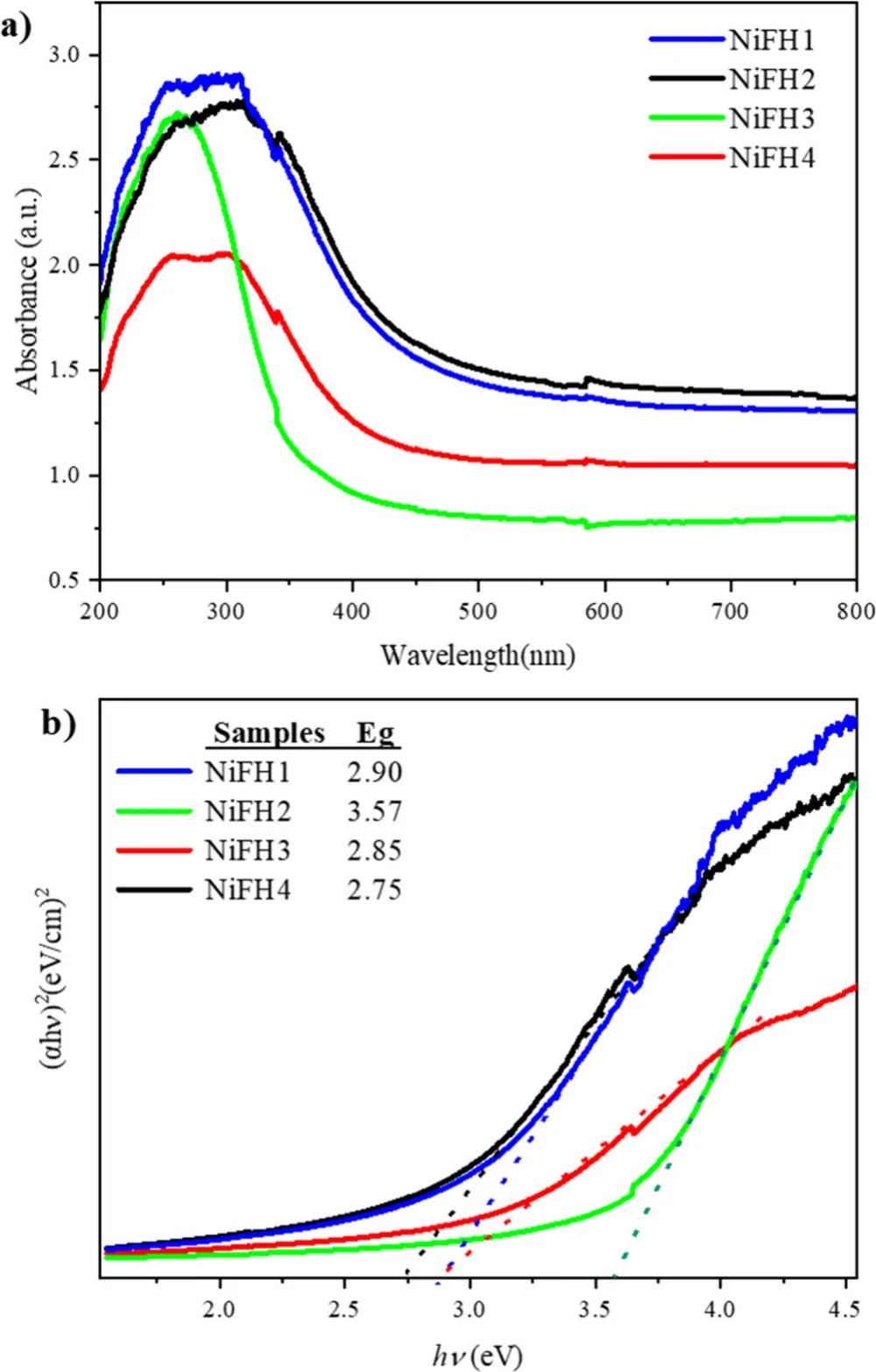
(a) Wavelength-dependent UV–vis absorption spectra and (b) Tauc plots used to determine the direct optical band gaps (*E*_*g*_^*d*^) of the composite hydrogels prepared with varying NiFe_2_O_4_–cellulose concentrations.

To further elucidate the optoelectronic characteristics of the materials, the direct optical band gap energies (*E*_*g*_^*d*^) were calculated using Tauc’s relation,^46,47^ as graphically illustrated in Figure 4b. By extrapolating the linear regions of the (*αhν*)^2^ versus photon energy (*hν*) plots to the energy axis, the direct band gap values were explicitly derived. Furthermore, utilizing the absorbance data, the indirect band gap (*E*_*g*_^*id*^) and the Urbach energy (*E*_*U*_)—which serves as a critical measure of structural disorder—were calculated for each sample. All extracted optical parameters (*E*_*g*_^*d*^, *E*_*g*_^*id*^, and *E*_*U*_) are comprehensively summarized in Table 1. The derived energy gap values fall well within the typical 0.1 to 4.0 eV range, providing strong evidence that the prepared biocomposite hydrogels exhibit distinct semiconductor characteristics.^48,49^

**Table 1:** Calculated Direct Optical Band Gap (*E*_*g*_^*d*^), Indirect Optical Band Gap (*E*_*g*_^*id*^), and Urbach Energy (*E*_*U*_) Values for the NiFe_2_O_4_–Cellulose Biocomposite Hydrogels

Energy	NiFH1	NiFH2	NiFH3	NiFH4
*E*_*g*_^*d*^	2.9	3.57	2.85	2.75
*E*_*g*_^*id*^	0.80	2.13	0.68	0.65
*E*_*u*_	1.79	1.10	1.82	1.74

A clear composition-dependent trend is observed among the optical parameters (Table 1). The NiFH2 sample displays the highest direct and indirect forbidden energy gaps (3.57 and 2.13 eV, respectively) alongside the lowest Urbach energy (1.10 eV). This compelling correlation leads to the conclusion that this specific optimal formulation possesses the most ordered structural network and the lowest defect density among the examined materials. In contrast, for the NiFH4 sample, which contains the highest nanoparticle doping ratio, the direct *E*_*g*_^*d*^ value decreases significantly to 2.75 eV, and the indirect *E*_*g*_^*id*^ drops to 0.65 eV. Concurrently, the *E*_*U*_ value increases to 1.74 eV.^50–52^ This elevated Urbach energy quantitatively confirms that higher nanoparticle concentrations intensify structural disorder at the polymer matrix–nanoparticle interfaces. Such interfacial perturbations lead to the generation of new localized energy states and the extension of band tails deep into the forbidden energy gap, which consequently results in the observed band gap narrowing. Furthermore, the observation that the direct band gap values are consistently greater than their indirect counterparts for all samples suggests that phonon assistance is required for momentum conservation during electronic transitions, thereby enabling electrons to be excited by lower-energy photons.^53–55^

### 3.4 Dielectric Properties

The frequency-dependent dielectric properties of the NiFe_2_O_4_-incorporated cellulose composite hydrogels were investigated across a broad frequency spectrum (10^1^–10^7^ Hz). Generally, the dielectric behavior of such nanocomposites is dictated by morphological features, nanoparticle size and surface area, alongside their interfacial interactions with the polymeric matrix.^56^ The frequency evolution of the real (*ε*′) and imaginary (*ε*″) parts of the complex dielectric permittivity, as well as the dielectric loss tangent (tan δ), are presented in detail in Figure 5a–c, respectively.

**Figure 5:**
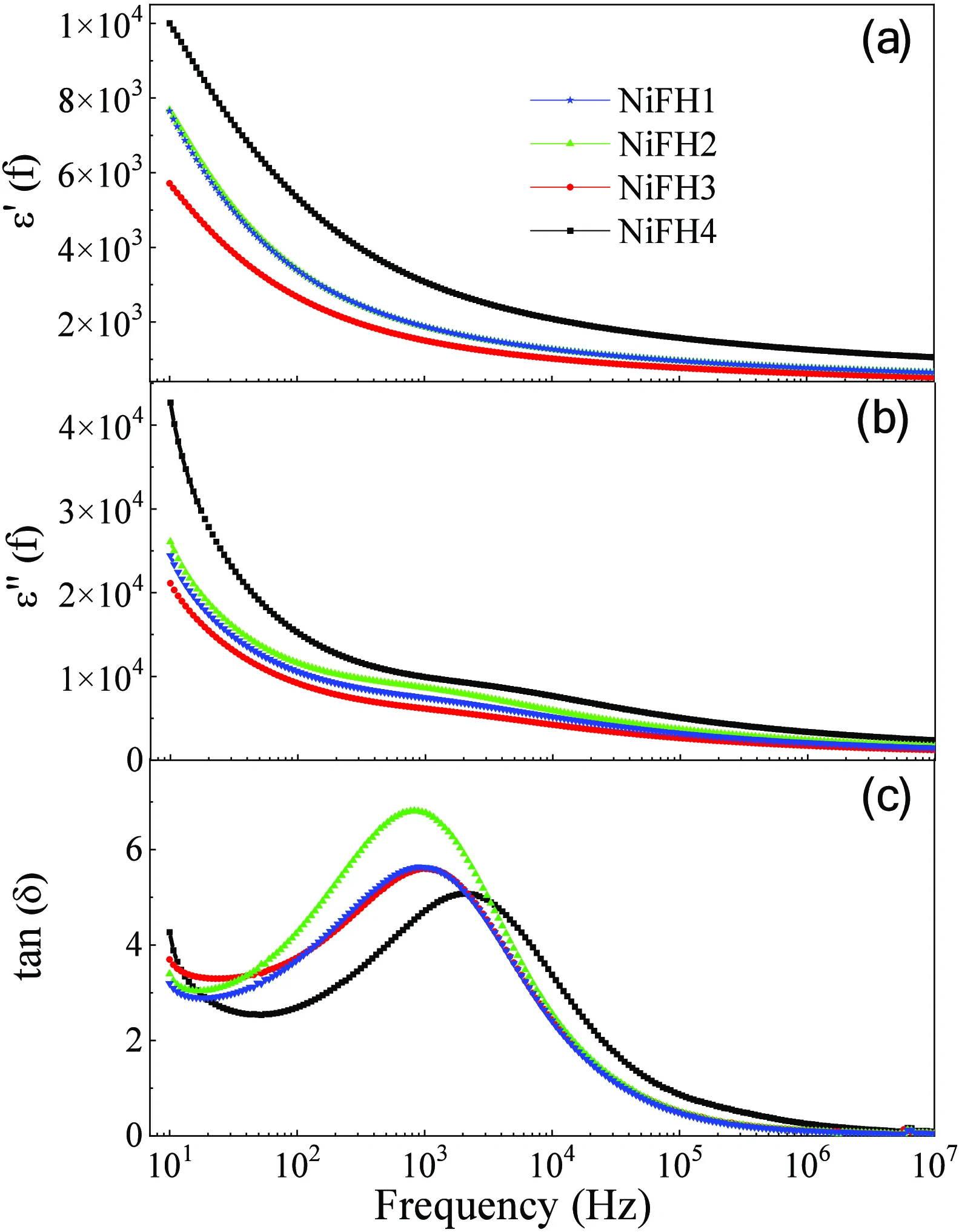
Frequency dependence of (a) the real part of the dielectric permittivity (*ε*′), (b) the imaginary part of the dielectric permittivity (*ε*″), and (c) the dielectric loss tangent (tan δ) for the NiFe_2_O_4_-doped biocomposite hydrogels at various nanoparticle concentrations.

Figure 5a illustrates the frequency dependence of *ε*′, which represents the material’s electrical energy storage capacity and reflects the extent of internal polarization. Across all samples, *ε*′ exhibits its maximum values in the low-frequency region. This pronounced low-frequency response is primarily attributed to Maxwell–Wagner interfacial polarization. At lower frequencies, the applied alternating electric field changes slowly enough to allow charge carriers ample time to migrate and dipoles to fully align, thereby activating all available polarization mechanisms.^57–59^ As the frequency increases, the dipoles can no longer keep pace with the rapid field oscillations. Consequently, their contribution to overall polarization diminishes, leading to an exponential drop in the *ε*′ value. This specific frequency-dependent decay aligns perfectly with Koops’ phenomenological theory, which is based on the Maxwell–Wagner two-layer model.^60^

Similarly, the imaginary part of the dielectric constant (*ε*″), which quantifies the energy dissipated as heat and elucidates the material’s conductivity mechanisms, exhibits a corresponding frequency-dependent decay (Figure 5b). The exceptionally high *ε*″ values observed in the low-frequency regime originate predominantly from ionic conductivity and the extended mobility of free charges migrating through the hydrogel network.^61^ In this regime, the slow oscillation of the electric field forces charge carriers to travel longer macroscopic distances, converting electrical energy into thermal energy via internal friction. Furthermore, according to the inverse proportional relationship between imaginary permittivity and angular frequency (*ε*″ ∝ σ/(*ε*_0_ω)), a decrease in frequency mathematically inherently drives the *ε*″ values upward, thereby explaining the sudden exponential increase at the tail of the graph.^62^ The substantial initial rise in both *ε*′ and *ε*″ is also indicative of an enormous accumulation of space charges at the macroscopic interface between the metallic electrode and the ion-rich doped hydrogel.^63^ In the mid-frequency range (10^2^–10^4^ Hz), a slight shoulder formation is discernible in the curves, which is characteristic of a dominant dielectric relaxation process occurring within the composite matrix. Overall, the observed frequency dependence of both permittivity components is in excellent agreement with the existing literature.^64–67^

An analysis of the compositional effect reveals a nonlinear relationship between the NiFe_2_O_4_ additive ratio and the resulting dielectric properties. While the samples containing 0.25% and 0.50% fillers exhibited comparable dielectric responses, a noticeable decrease was observed at the 0.75% doping level. Conversely, increasing the additive concentration to 1.0% (NiFH4) resulted in the dielectric parameters reaching their maximum critical threshold values. This enhancement at the highest loading level can be attributed to the strengthening of interfacial polarization mechanisms, the formation of more robust bonds, and the facilitated, stable migration of free ions within the percolated hydrogel network.^68–70^

The dielectric loss tangent (tan δ = *ε*″/*ε*′), representing the ratio of dissipated energy to stored energy, is depicted in Figure 5c. This parameter is critically important for evaluating material performance in insulation applications, energy storage systems, and dielectric resonance devices against microwaves.^68,71^ Because the *ε*″ values in the low-frequency regime are approximately four times greater than the corresponding *ε*′ values across all concentrations, the initial tan δ values remain relatively high. Specifically, materials exhibiting robust electromagnetic wave absorption capabilities are expected to possess high loss tangent values (tan δ > 1), signifying significant degradation of the incident electrical energy.^72^

In the mid-frequency range, distinct relaxation peaks appear in the tan δ spectra. These peaks indicate the specific frequencies where energy loss reaches its maximum and relaxation mechanisms are most active. The variations in the height and positional frequency of these peaks, which correspond to shifts in the relaxation time (τ), are directly dictated by the filler concentration, the density of the nanoparticle–polymer interfaces, and altered ion mobility.^73^

Finally, in the high-frequency region, both the permittivity components (*ε*′ and *ε*″) and the loss tangent decay to minimal values and exhibit frequency-independent behavior. This plateau is attributed to the ultimate weakening of the dielectric response; the rapid field oscillations severely restrict ion movement and dipole orientation, effectively paralyzing the relaxation and energy dispersion processes within the doped hydrogels.^63,74,75^

### 3.5 Complex Impedance Spectroscopy

To elucidate the charge transport mechanisms and interfacial polarization dynamics, the complex impedance parameters of the composite hydrogels were analyzed across a broad frequency spectrum (Figure 6). The complex impedance is mathematically expressed as Z* = *Z*′ – *jZ*″, where the real component (*Z*′) represents the resistance and the imaginary component (*Z*″) represents the reactance. Impedance spectroscopy is a crucial analytical tool that elucidates the internal resistance encountered during the transport of ions and charge carriers within a material’s microstructural network.^76,77^ The experimental results reveal that the synergistic interactions between the conductive NiFe_2_O_4_ nanoparticles and the dielectric hydrogel matrix create a multiphase architecture characterized by distinct interfacial regions.

**Figure 6:**
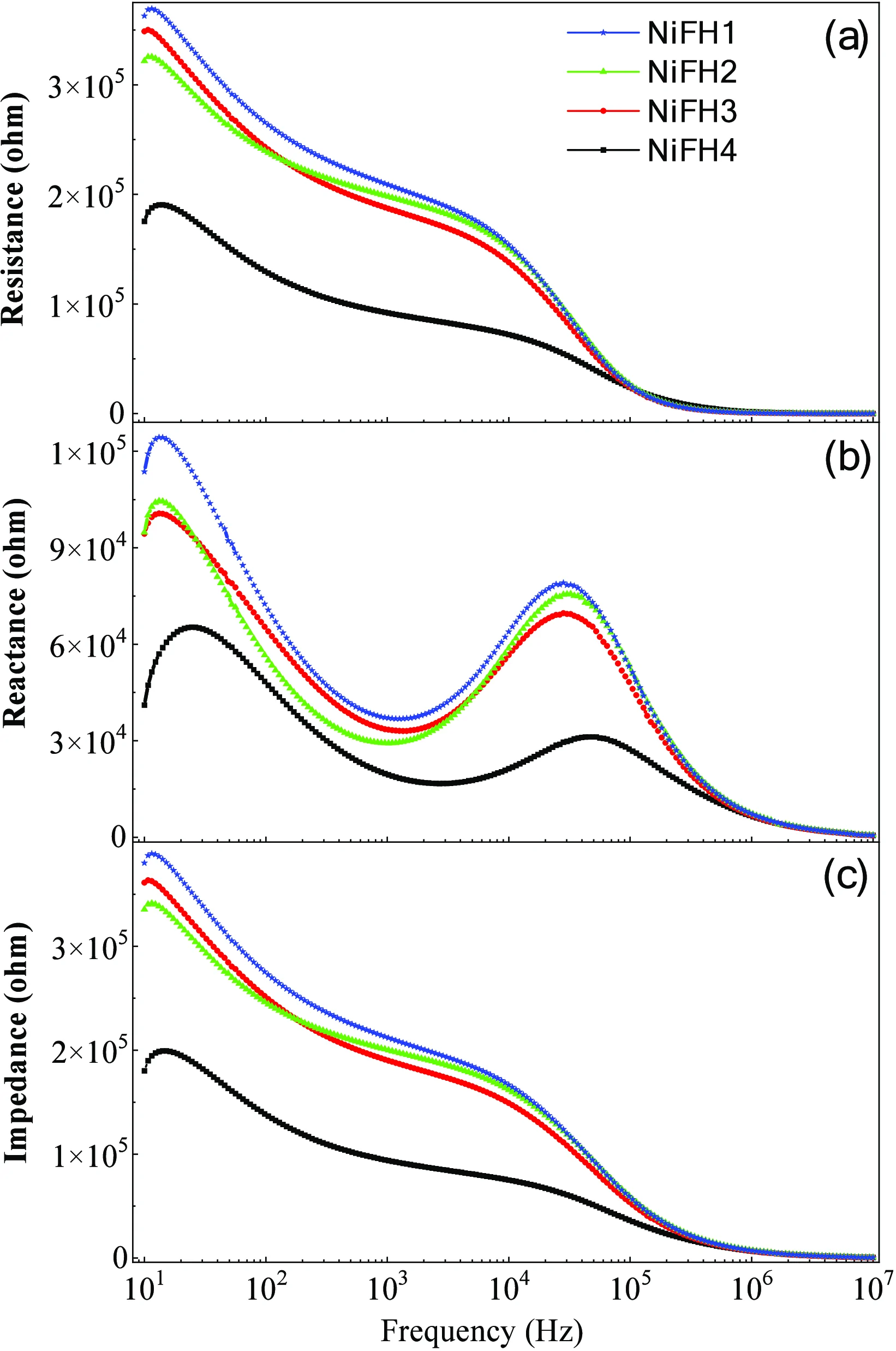
Frequency dispersion of (a) resistance (*Z*′), (b) reactance (*Z*″), and (c) total impedance magnitude (|*Z*|) for the NiFe_2_O_4_–cellulose biocomposite hydrogels with varying filler concentrations.

Figure 6a displays the frequency dispersion of the real part of the impedance (resistance, *Z*′). At low frequencies, carrier mobility is severely restricted, resulting in high *Z*′ values. This elevated resistance is primarily governed by Maxwell–​Wagner–Sillars (MWS) interfacial polarization; in this regime, charge carriers accumulate at the grain boundaries and polymer–nanoparticle interfaces, thereby creating substantial internal resistive barriers.^78,79^ As the applied frequency increases, the alternating electric field oscillates too rapidly for space charges to accumulate at these interfaces. Consequently, trapped carriers are liberated to move more freely, causing the observed continuous and accelerated decrease in the *Z*′ value.^80–83^ Above 10^5^ Hz, the rate of decrease diminishes significantly, and the resistance values plateau to a nearly constant minimum across all samples. This high-frequency convergence occurs due to the complete disappearance of electrode and interfacial polarization effects, leaving only the intrinsic bulk resistance of the material active.

The dynamic electrical responses of the hydrogels were further characterized by analyzing the imaginary part of the impedance (*Z*″, reactance), as depicted in Figure 6b. The reactance spectra clearly exhibit two distinct dynamic features: a low-frequency shoulder and a prominent high-frequency peak. The presence of these two separate relaxation regions strongly suggests that the alternating current (AC) electrical behavior of the composite hydrogel system can be modeled by two equivalent RC circuits operating in series,^29,84–86^ a hypothesis that will be quantitatively verified through the complex impedance plane analysis in the subsequent section.

Figure 6c illustrates the variation of the total impedance magnitude (|*Z*|), which is a comprehensive parameter formed by the vector sum of the *Z*′ and *Z*″ components. Because the real component (*Z*′) is significantly larger than the reactive component (*Z*″) across the entire frequency spectrum, the overall impedance profile closely mirrors the resistive behavior of *Z*′. Even though slight deviations occur near the *Z*″ relaxation peaks, the total impedance curve aligns strictly with *Z*′ since it is the dominant component of the system.^87^

Regarding the effect of filler concentration, a clear inverse relationship is observed between the NiFe_2_O_4_ loading and the impedance magnitude. As shown in the comparative spectra, the NiFH1 sample (with the lowest concentration) exhibits the highest impedance values, whereas the NiFH4 sample (highest concentration) displays the lowest impedance. The reduction in impedance in the highly doped sample indicates an enhancement in polarization quality, coupled with more highly polarized electrode–material interfaces.^82,83,88^ More importantly, this phenomenon can be attributed to the reduction in the interparticle distances as the nanoparticle concentration increases. The closer proximity of the NiFe_2_O_4_ nanoparticles within the hydrogel matrix facilitates charge transfer mechanisms, such as electron hopping or quantum tunneling, between neighboring conductive sites. Consequently, this enhanced charge mobility reduces the overall internal resistance of the composite, leading to the macroscopic decrease observed in total impedance.^89^ Furthermore, the Cole–Cole impedance response of all samples measured after approximately one year closely matched the initial measurements, demonstrating the long-term stability and measurement reproducibility of the hydrogels (Figure S3).

The complex impedance behavior (Z* = *Z*′ + *jZ*″) of the NiFe_2_O_4_-doped cellulose-supported hydrogels was comprehensively analyzed using Smith chart representations, as depicted in Figure 7. Serving as a powerful tool for visualizing impedance dynamics, the Smith chart enables the real and imaginary components of complex impedance to be plotted clearly within a single complex plane.^90,91^ The charts exhibit two distinct semicircular arcs for all samples, indicating that the overall impedance is governed by two major microstructural contributions: the grain boundaries (including interfacial and electrode polarizations) dominating the low-frequency region and the bulk material properties characterizing the high-frequency response.^92–96^ Notably, with the exception of the composite containing the highest nanoparticle loading (NiFH4), the complex impedance trajectories of the samples display highly similar behavior. Consequently, the total impedance can be modeled as a series combination of these components, mathematically expressed as Z*­(*f*) = *Z*_gb_^*^(*f*) + *Z*_b_^*^(*f*).^97^

**Figure 7:**
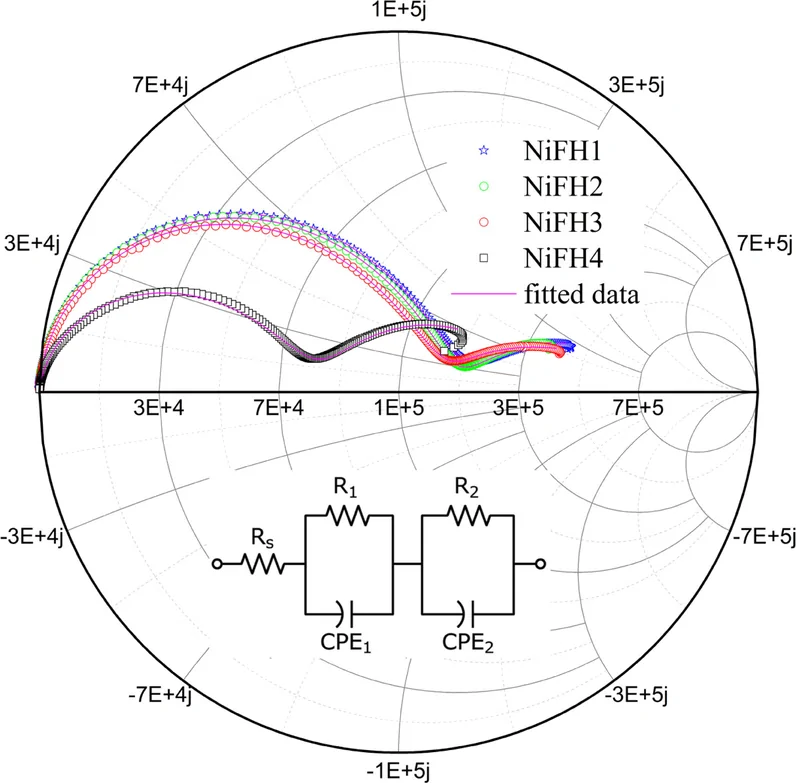
Complex impedance plane (Smith chart) plots and corresponding equivalent circuit fits for the NiFe_2_O_4_-doped biocomposite hydrogels at various concentrations.

The visibly depressed nature of these semicircular arcs at high frequencies indicates a distinct deviation from the ideal Debye relaxation model, which assumes a single uniform relaxation time.^35^ Because this characteristic behavior stems from the heterogeneous nature of the composite and its distributed relaxation times, the Cole–Cole model incorporating constant phase elements (CPEs) provides a much more accurate physical explanation than ideal capacitors. The empirical Cole–Cole impedance is defined as Z* = *R*_∞_ + $\frac{R_{0}-R_{\infty }}{1+{\left(i\omega \tau \right)}^{n}}$ , where *R*_0_ and *R*_∞_ represent the low- and high-frequency resistances, respectively, and *n* (0 < *n* ≤ 1) is the constant phase exponent. Correspondingly, the impedance of the CPE is defined as *Z*_*CPE*_ = 1/[*Q*(*iω*)^*n*^].^95,96^

To quantitatively extract these physical parameters, the experimental data were fitted to the equivalent electrical circuit model (inset of Figure 7) using the EIS Spectrum Analyzer software. The fitting process employed the Powell algorithm with an unweighted approach over 600 iterations. The theoretically fitted curves exhibit excellent agreement with the experimental Smith chart data, and the derived equivalent circuit parameters are summarized in Table 2. In the proposed model, the *R*_1_ and CPE_1_ components represent the grain boundary and interfacial contributions dominant in the low-frequency region, whereas the *R*_2_ and CPE_2_ pair characterize the bulk conduction mechanisms effective at high frequencies.^29^ Furthermore, *R*_*s*_ denotes the series resistance originating from the external measurement system (cables and contacts). Determined from the high-frequency intercept on the real axis, *R*_*s*_ remains negligible compared to the massive internal resistances of the material.^98–100^

**Table 2:** Equivalent Electrical Circuit Parameters Extracted from the Impedance Spectra of the Biocomposite Hydrogels at Various NiFe_2_O_4_ Concentrations

Samples	*R*_*s*_ (Ω) × 10^–13^	*R*_1_ (Ω) × 10^6^	*R*_2_ (Ω) × 10^5^	CPE_1_ *×* 10^–7^	*n*_1_	CPE_2_ *×* 10^–10^	*n*_2_
NiFH1	0.48	1.00	1.82	3.81	0.484	1.47	0.874
NiFH2	4.02	1.00	1.80	4.86	0.496	1.50	0.869
NiFH3	3.46	0.64	1.58	4.08	0.469	1.51	0.880
NiFH4	1.46	0.41	0.75	4.27	0.523	3.50	0.832

An analysis of the resistive components (Table 2) reveals that the *R*_1_ values are significantly larger than the *R*_2_ values across all samples, proving that charge carriers encounter the primary impedance barriers at the interfaces and grain boundaries rather than within the bulk. As the NiFe_2_O_4_ content increases, a systematic decrease in both resistance components is observed, demonstrating that the nanoparticles simultaneously enhance both bulk and interfacial conductivity.^86^ Evaluating the CPE exponents, which characterize the system’s capacitive behavior, the low-frequency *n*_1_ values (≈0.48–0.52) reflect highly nonideal capacitive behavior driven by strong interfacial polarization and diffusion effects. Conversely, the high-frequency *n*_2_ values (≈0.83–0.88) indicate that the bulk system exhibits a stable dielectric character that approaches ideal capacitive behavior (*n* → 1).^101^ The fitting reliability was confirmed by the calculated error margins, which were restricted to 2.8%–12.7% for *R*_1_ and deemed entirely negligible for the other parameters due to their minimal values.

### 3.6 AC Conductivity Analysis

The frequency-dependent AC conductivity (σ_*ac*_) of the NiFe_2_O_4_-doped biocomposite hydrogels is presented in Figure 8. The conductivity spectra reveal that the electrical transport properties are governed by frequency-dependent mechanisms, distinctly categorized into three dynamic phases: a low-frequency dispersion (Region I), a mid-frequency plateau (Region II), and a high-frequency linear dispersion (Region III).^102^ The dynamic response of σ_*ac*_, which is fundamentally linked to the dielectric loss via the relation σ_*ac*_ = ω*ε*_0_ *ε*″, can be comprehensively modeled using Jonscher’s universal power law (σ_*ac*_ = σ_*dc*_ + *A*ω^*s*^).^103^ In this empirical expression, σ_*dc*_ represents the frequency-independent direct current conductivity, *A* is a pre-exponential constant defining the polarization strength, and *s* (0 < *s* ≤ 1) is the dimensionless exponent characterizing the specific conduction mechanism of the material.^104^

**Figure 8:**
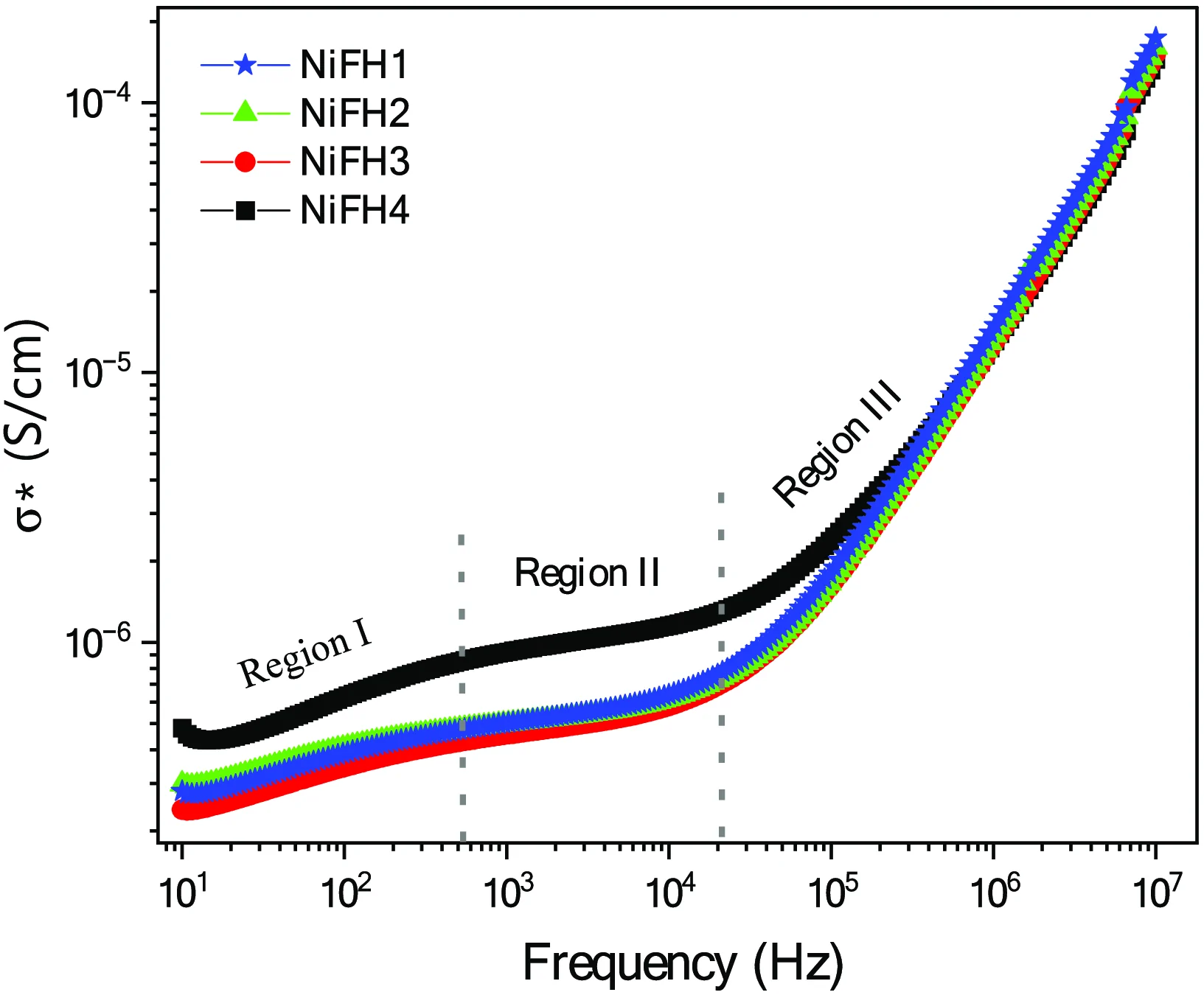
Frequency dependence of the AC conductivity (σ_*ac*_) for the NiFe_2_O_4_-doped biocomposite hydrogels.

In the low-frequency regime, minor frequency dispersion is observed, primarily induced by electrode polarization and interfacial space-charge accumulation. As the frequency progresses into the mid-frequency range, the conductivity stabilizes into a prominent, frequency-independent plateau. This plateau exactly corresponds to the σ_*dc*_ term in Jonscher’s law, representing the regime where the long-range translational motion of free charge carriers becomes dominant, and the macroscopic transport is predominantly regulated by the resistive grain boundaries.^105–107^

Upon transitioning to the high-frequency regime (>10^4^ Hz), the conductivity exhibits a sharp, linear, and dispersive increase, seamlessly aligning with the frequency-dependent *Aω* ^*s*^ component of Jonscher’s law. This substantial rise is attributed to the intensification of short-range hopping motions of charge carriers between localized states within the composite network. At these elevated frequencies, the rapidly oscillating electric field effectively releases the space charges previously trapped at the interfaces, thereby eliminating internal conduction barriers. Furthermore, thermal fluctuations within the lattice structure enhance the probability of charge carriers tunneling across internal energy barriers, contributing significantly to the elevated macroscopic conductivity.^106,108^

This dynamic transition from boundary-dominated resistance at low frequencies to grain-assisted short-range conduction at high frequencies is in complete agreement with Koops’ phenomenological theory.^60^ Finally, an evaluation of the compositional effect reveals that the NiFH4 sample exhibits superior conductivity performance, particularly across Regions I and II. The establishment of this enhanced conductive network underscores the distinct advantage of the highly doped composite for implementation in advanced electronic and charge transport applications compared to its low-concentration counterparts.

### 3.7 Capacitive Behavior and Energy Storage Performance

The frequency-dependent parallel capacitance (*C*_*p*_) of the NiFe_2_O_4_-doped biocomposite hydrogels is presented in Figure 9. As anticipated from the fundamental relationship between capacitance and polarizability, the *C*_*p*_ spectra exhibit an evolutionary trend that is in complete agreement with the real dielectric permittivity (*ε*′) data discussed previously (Figure 5a). At low frequencies, the capacitance reaches its maximum value across all samples. This robust charge storage capacity is primarily driven by Maxwell–​Wagner–Sillars interfacial polarization and the massive accumulation of free charges at the heterogeneous boundaries.^35,109–112^ The incorporation of the magnetic nanofillers effectively modifies the interfacial interactions within the polymer matrix, creating a higher density of polarizable sites and thereby significantly enhancing the overall macroscopic polarization.^113^

**Figure 9:**
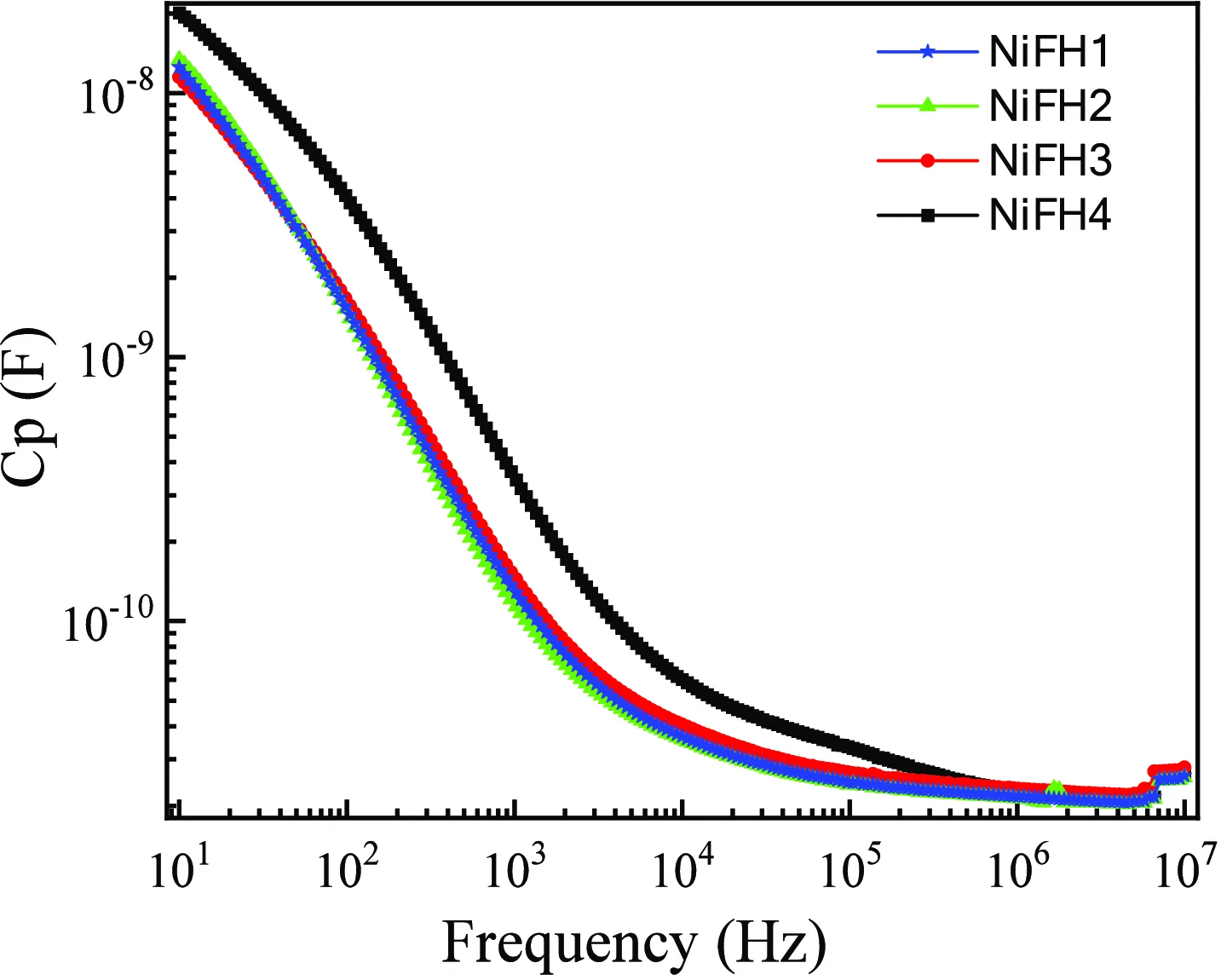
Frequency dependence of the parallel capacitance (*C*_*p*_) for the biocomposite hydrogels at various NiFe_2_O_4_ nanoparticle concentrations.

Evaluating this capacitive response via electrochemical impedance spectroscopy (EIS) is particularly crucial for the biological integration of these materials. Specifically, understanding low-frequency capacitance and ion migration dynamics provides critical insights for the deployment of such hydrogels in biomedical applications, biocompatible interfaces, and advanced biosensors.^114^ Among the investigated formulations, the highly doped NiFH4 composite consistently exhibits the maximum capacitance across the entire measured frequency spectrum. This superior capacitive performance is a direct consequence of the expanded electroactive interfacial area and the intensified polarization density resulting from the increased NiFe_2_O_4_ nanoparticle concentration.

As the applied frequency increases, the *C*_*p*_ values undergo a characteristic decay due to typical dielectric relaxation phenomena, eventually plateauing at high frequencies where dipoles and charge carriers can no longer track the rapidly oscillating electric field.^115–117^ Ultimately, the exceptional low-frequency charge storage capacity and the stable dielectric relaxation demonstrated by the NiFH4 composite underscore its significant potential as a high-performance functional material for low-frequency energy storage systems and advanced bioelectronic devices.

## 4 Conclusion

This research successfully established a systematic framework for developing sustainable optoelectronic materials by integrating hemp-derived cellulose into a NiFe_2_O_4_-doped poly­(AMPS/IA) hydrogel matrix. The synergistic integration of these components was demonstrated through a multiscale characterization approach, where XRD, SEM, and STEM collectively confirmed a densely interconnected amorphous network.

The STEM images further indicated that the magnetic nanoparticles are dispersed throughout the matrix without severe microscale agglomeration, while the retention of the spinel NiFe_2_O_4_ phase was independently supported by the characteristic metal–oxygen bands in the FTIR analysis. Thermogravimetric analysis further confirmed that the stepwise integration of NiFe_2_O_4_ and the poly­(AMPS/IA) network markedly improved the thermal stability and structural integrity of the composite relative to its precursors.

Optical analyses revealed that the synthesized biocomposites exhibit highly tunable semiconductor characteristics, with direct band gap energies (*E*_*g*_^*d*^) modulated between 2.75 and 3.57 eV. While the NiFH2 formulation showed the highest structural order (*E*_*U*_ = 1.10 eV), increasing the nanoparticle loading to 1.0% (NiFH4) induced significant localized structural disorder at the polymer–filler interfaces. This phenomenon, quantitatively corroborated by the rise in Urbach energy to 1.74 eV, led to the broadening of band tails and a systematic narrowing of the band gap. Consequently, the material’s optical response was effectively tuned from a wide-band-gap state toward a more pronounced semiconducting regime.

Our broadband dielectric results revealed that the polarization characterization of all biocomposite samples was perfectly consistent with Maxwell–Wagner interfacial polarization in the low-frequency region and Koops’ phenomenological theory in the high-frequency region. It was observed that the NiFe_2_O_4_ concentration was the decisive factor dictating the dielectric characteristics; the composite hydrogel with the highest dopant loading (NiFH4) exhibited the maximum values for dielectric parameters governing stored and dissipated energy. It was concluded that the samples demonstrated massive capacitive effects due to facilitated interfacial polarization at low frequencies, making them highly suitable for use as energy storage circuit elements operating in the low-frequency regime.

Furthermore, the complex impedance plane plots, which modeled the AC electrical behavior of the samples, were found to be strictly equivalent to two series-connected RC circuits on the Smith chart. Crucially, this modeling revealed that grain boundaries dominate the low-frequency region, while bulk material properties characterize the high-frequency response. The analysis explicitly showed that the interfacial resistance (*R*_1_) is significantly larger than the bulk resistance (*R*_2_), proving that charge carriers encounter their primary impedance barriers at the interfaces rather than within the bulk. Moreover, as the NiFe_2_O_4_ content increased, a systematic decrease in both resistance components was observed, demonstrating that the nanoparticles simultaneously enhance both bulk and interfacial conductivity. Through this mechanism, the composite with the highest NiFe_2_O_4_ amount was confirmed to possess the highest overall AC conductivity, driven by enhanced electron hopping and quantum tunneling.

Ultimately, based on the highly promising and tunable optical and dielectric properties observed, along with their inherent mechanical flexibility, it was concluded that these NiFe_2_O_4_-doped cellulose/poly­(AMPS/IA) composite hydrogels possess massive potential as functional circuit components for versatile frequency applications in green and flexible electronics, where the precise charge transfer capabilities can be effectively modulated by the biocomposite dopant amount.

## Supplementary Material



## Data Availability

The data sets generated and analyzed during the current study are available from the corresponding author on reasonable request.

## Notes

EDS spectra of the NiFe_2_O_4_–cellulose precursor and the NiFH4 biocomposite hydrogel; UV–vis absorbance spectra of the NiFH1–NiFH4 biocomposite hydrogels recorded approximately one year apart; and Cole–Cole (complex impedance plane) plots of the NiFH1–NiFH4 biocomposite hydrogels recorded approximately one year apart (PDF)
