# Supplementary material for: Hemp-Derived Cellulose/NiFe2O4 Biocomposite Hydrogels for Flexible Optoelectronics
Source: ACS Omega. 2026 Oct 7;11(41):62852–65. doi: 10.1021/acsomega.6c04724 (PMC13689293; doi:10.1021/acsomega.6c04724)
Supplement: Supplementary file 1 [file ao6c04724_si_001.pdf]

Supporting Information

Hemp-derived cellulose/$NiFe_2O_4$ bio-composite hydrogels for flexible optoelectronics

Orhan Yalçın [a,*], Ahmed.M.J. Al-dala Ali[b], Ramazan Coşkun[c], Hasan Uslu[d]

[a]*Faculty of Sciences, Department of Physics, Nigde Omer Halisdemir University, 51240 Niğde, Turkiye;*
[b]*Institute of Science, Niğde Ömer Halisdemir University, 51240, Niğde, Türkiye*
[c]*Department of Chemistry, Yozgat Bozok University, Yozgat 66500, Türkiye*
[d]*Department of Food Engineering, Niğde Ömer Halisdemir University, 51240, Niğde, Türkiye*

****Corresponding author***.

E-mail address: o.yalcin@ohu.edu.tr δ orhanyalcin@gmail.com (Orhan Yalçın).

Table of Contents

Figure S1. EDS spectra of (a) the $NiFe_2O_4$–cellulose precursor and (b) the NiFH4 bio-composite hydrogel.

Figure S2. UV–Vis absorbance spectra of the NiFH1–NiFH4 bio-composite hydrogels measured (a) approximately one year earlier and (b) during the revision.

Figure S3. Cole–Cole (complex impedance plane) plots of the NiFH1–NiFH4 bio-composite hydrogels measured (a) approximately one year earlier and (b) during the revision.

Figure S1a shows the EDS spectrum of the $NiFe_2O_4$–cellulose precursor, that is, the material prior to its incorporation into the hydrogel network. The Fe Kα (6.40 keV) and Ni Kα (7.48 keV) emission lines are clearly resolved above the bremsstrahlung continuum, with quantification giving 12.27 wt % Fe and 8.09 wt % Ni at relative uncertainties below 1 %, establishing the identity and composition of the ferrite-bearing phase at this stage of the synthesis.

Figure S1b shows the corresponding spectrum of the NiFH4 bio-composite hydrogel, displaying the characteristic emission lines of the constituent elements (C, N, O, S, Fe, and Ni). The precursor is incorporated at concentrations of 0.25–1.0 % (w/v) across the NiFH1–NiFH4 series, and the ferrite constitutes only around one quarter of the precursor powder; even in NiFH4, the sample of highest loading, the Fe and Ni contents of the hydrogel are therefore of the order of 0.2–0.3 wt % and 0.1–0.2 wt % respectively. These values lie at or below the practical detection limit of EDS for a millimetre-thick specimen composed almost entirely of light elements, where the bremsstrahlung background from the organic matrix is high and absorption effects are severe, and the Fe Kα and Ni Kα lines are accordingly not resolved above the background in this spectrum. Figure S1b is therefore presented as the elemental profile of the organic matrix rather than as evidence for the presence of the Fe- and Ni-bearing phase in the final composite; the identity and composition of that phase are established independently in Figure S1a.

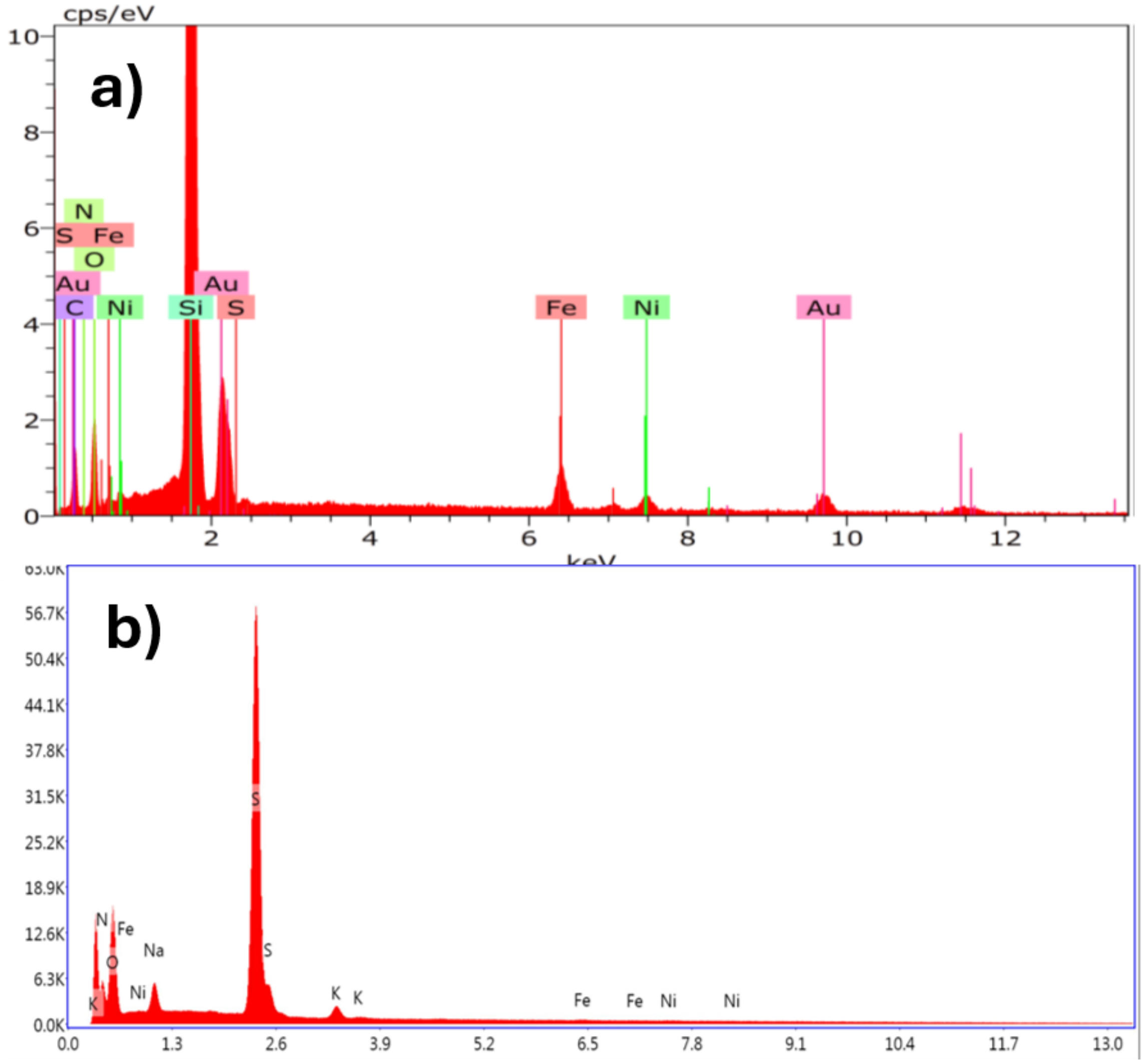

Figure S1. EDS spectra of (a) the $NiFe_2O_4$–cellulose precursor, in which the Fe Kα and Ni Kα lines are clearly resolved above the background, and (b) the NiFH4 bio-composite hydrogel, in which the Fe and Ni contents lie at or below the practical EDS detection limit for this specimen.

Long-term stability and reproducibility

To evaluate the long-term stability and reproducibility of the bio-composite hydrogels, the optical and impedance measurements performed during the revision were compared with those acquired approximately one year earlier on the same samples.

Optical stability. Figure S2 compares the UV–Vis absorbance spectra of the NiFH1–NiFH4 samples recorded (a) approximately one year earlier and (b) during the revision. The spectral profiles, absorption edges, and relative intensities remain unchanged over this period, confirming that the optical response of the materials is stable and the measurements are reproducible.

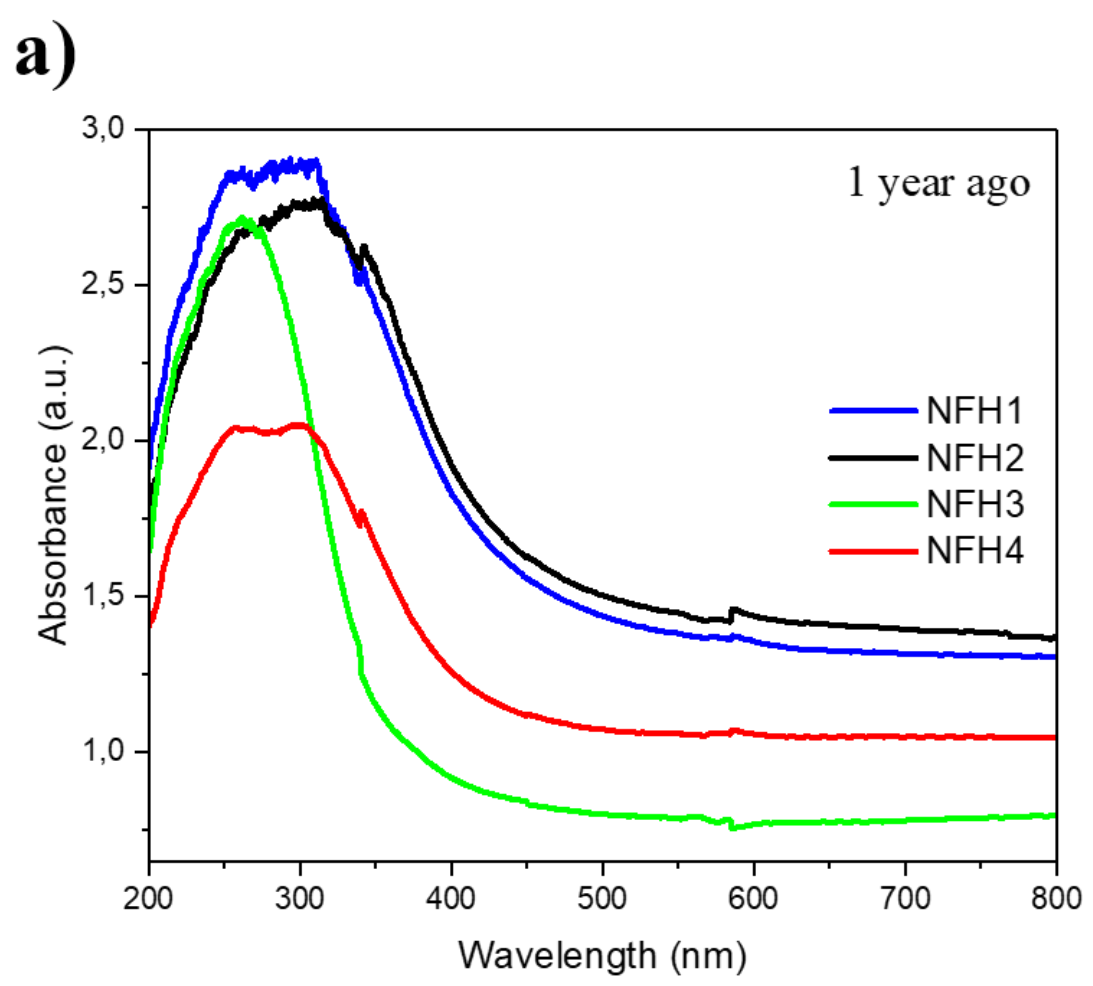

Figure S2. UV–Vis absorbance spectra of the NiFH1–NiFH4 bio-composite hydrogels measured (a) approximately one year earlier and (b) during the revision.

Figure S3 presents the corresponding Cole–Cole (complex impedance plane, Z′ vs. Z″) plots for the same samples recorded (a) approximately one year earlier and (b) during the revision. The two datasets exhibit closely matching arc shapes and impedance magnitudes, demonstrating that the charge-transport and interfacial-polarization behavior of the hydrogels remain stable over time. Together, the optical and impedance results confirm the long-term structural stability and measurement reproducibility of the $NiFe_2O_4$-doped bio-composite hydrogels.

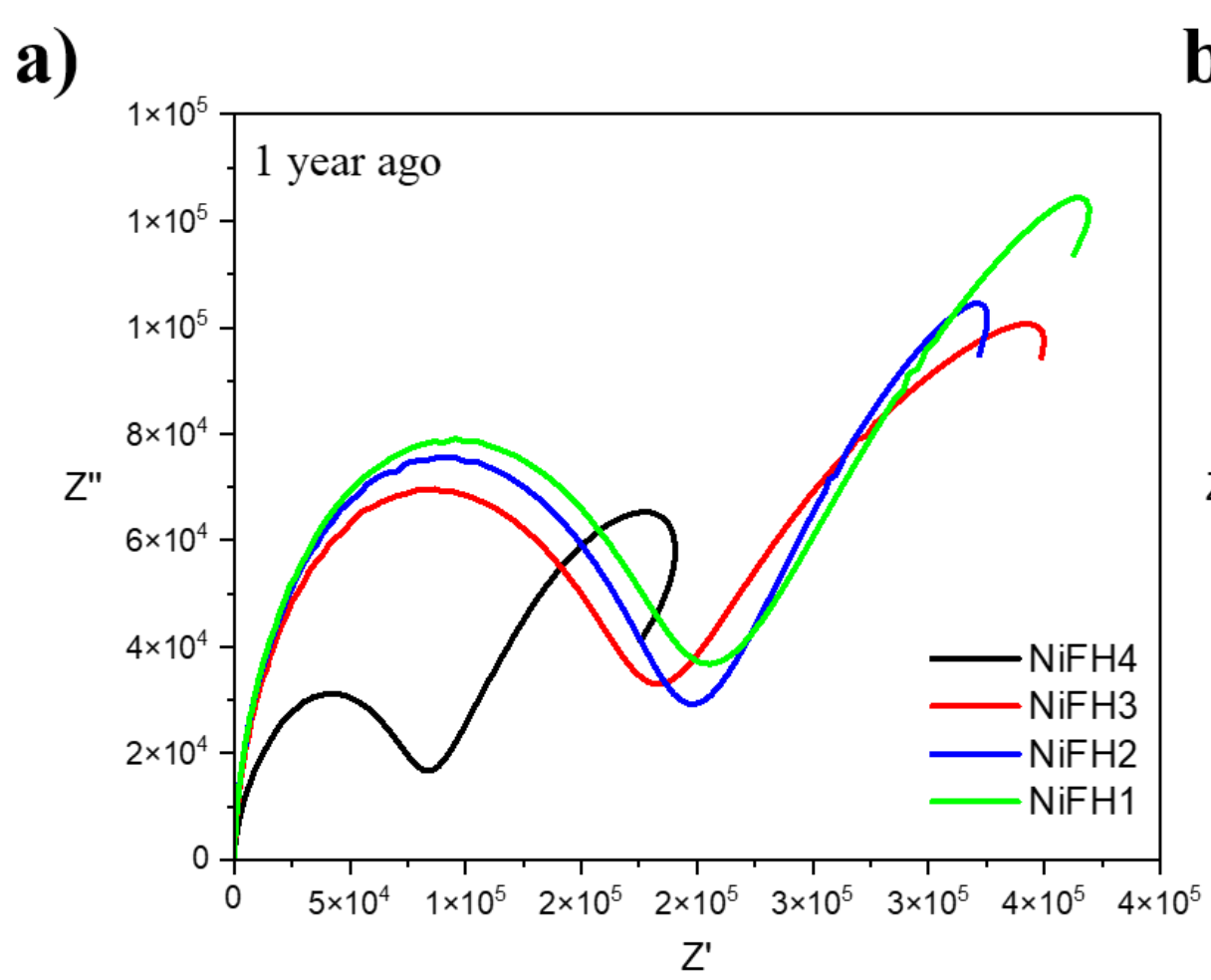

Figure S3. Cole–Cole (complex impedance plane) plots of the NiFH1–NiFH4 bio-composite hydrogels measured (a) approximately one year earlier and (b) during the revision.
